# An Unusual Cause of Small Bowel Obstruction in a Young Adult: A Case Report of a Patent Omphalomesenteric Duct

**DOI:** 10.7759/cureus.90235

**Published:** 2025-08-16

**Authors:** Mohamed Alatrash, Aya M Alamrawy

**Affiliations:** 1 General Surgery, Maidstone and Tunbridge Wells Hospital, Tunbridge Wells, GBR; 2 General and Colorectal Surgery, Cairo University, Cairo, EGY; 3 Breast Surgery, Guy's and St Thomas' NHS Foundation Trust, London, GBR; 4 General Surgery, Cairo University, Cairo, EGY

**Keywords:** diagnostic laparoscopy, embryologic, patent omphalomesenteric duct, small bowel obstruction, surgical case report

## Abstract

The omphalomesenteric duct is an embryologic structure that typically obliterates during early fetal development. Its persistence into adulthood is rare and may present with atypical clinical scenarios. We report a case of a 26-year-old male who presented with right lower quadrant abdominal pain initially suggestive of acute appendicitis. Intraoperative findings revealed a patent omphalomesenteric duct causing small bowel obstruction. The duct was resected, and a prophylactic appendectomy was performed. The patient recovered uneventfully and was discharged on postoperative day three without complications. Diagnosing the cause of intestinal obstruction in patients with no prior surgical history can be particularly challenging. Early recognition and timely surgical intervention in cases involving rare anomalies, such as a patent vitellointestinal duct, are critical. Surgeons should maintain a high index of suspicion for congenital causes in young adults presenting with bowel obstruction, as prompt management can significantly improve outcomes.

## Introduction

A patent omphalomesenteric duct is a very unusual anomaly that occurs in 2% of the population [1]. Clinically, persistent patent omphalomesenteric duct anomalies can remain asymptomatic or present with a variety of manifestations. In infancy and early childhood, they often cause symptoms such as abdominal pain, intestinal obstruction, gastrointestinal bleeding (e.g., melena due to ectopic gastric mucosa in a Meckel’s diverticulum), or umbilical drainage and hernia [2,3]. However, these malformations seldom cause symptoms in adulthood, and many remain incidental findings [4].

Indeed, most symptomatic cases present before the age of four years, and vitelline duct remnants are often quiescent in adults [4]. In the adult population, symptomatic omphalomesenteric duct remnants are exceedingly rare, and their diverse, nonspecific presentations can make preoperative diagnosis challenging [5]. When an adult patient with no history of abdominal surgery presents with intestinal obstruction, surgeons should keep uncommon congenital causes like a persistent omphalomesenteric duct in mind, as the diagnosis might only be established intraoperatively [6,7].

Prompt recognition and surgical management of such anomalies are important to prevent serious complications. We present a case of a young adult who arrived with clinical features mimicking acute appendicitis. Diagnostic laparoscopy revealed a patent vitellointestinal duct causing small bowel obstruction. This case underscores an uncommon etiology of intestinal obstruction in adults and was successfully managed at Tunbridge Wells Hospital, a district general hospital in the UK.

## Case presentation

A previously healthy 26-year-old male presented with a two-day history of worsening abdominal pain localized to the right iliac fossa, associated with several episodes of vomiting. On examination, he was afebrile and had localized tenderness in the right lower quadrant without significant abdominal distension. Bowel sounds were normal, and no palpable mass or hernia was noted. Laboratory investigations revealed mildly elevated inflammatory markers (C-reactive protein and white blood cell count), as shown in Table 1. An abdominal ultrasound was inconclusive, showing no definitive appendix, a small amount of free fluid in the right iliac fossa, and mildly dilated bowel loops in that region. These findings were concerning but not diagnostic.

**Table 1:** Laboratory results on admission.

Parameter	Value	Reference range
C-reactive protein (CRP)	6 mg/L	<5 mg/L
White blood cell (WBC) count	13.5 × 10⁹/L	4 – 11 × 10⁹/L
Hemoglobin (Hb)	152 g/L	130 – 170 g/L
Serum amylase	67 U/L	30 – 110 U/L
Total bilirubin	13 µmol/L	5 – 21 µmol/L
Alkaline phosphatase (ALP)	69 U/L	30 – 120 U/L
Alanine transaminase (ALT)	13 U/L	7 – 56 U/L
Sodium (Na⁺)	139 mmol/L	135 – 145 mmol/L
Potassium (K⁺)	4.4 mmol/L	3.5 – 5.0 mmol/L
Serum creatinine	97 µmol/L	60 – 110 µmol/L
Blood urea	6.9 mmol/L	2.5 – 7.8 mmol/L

**Figure 1:**
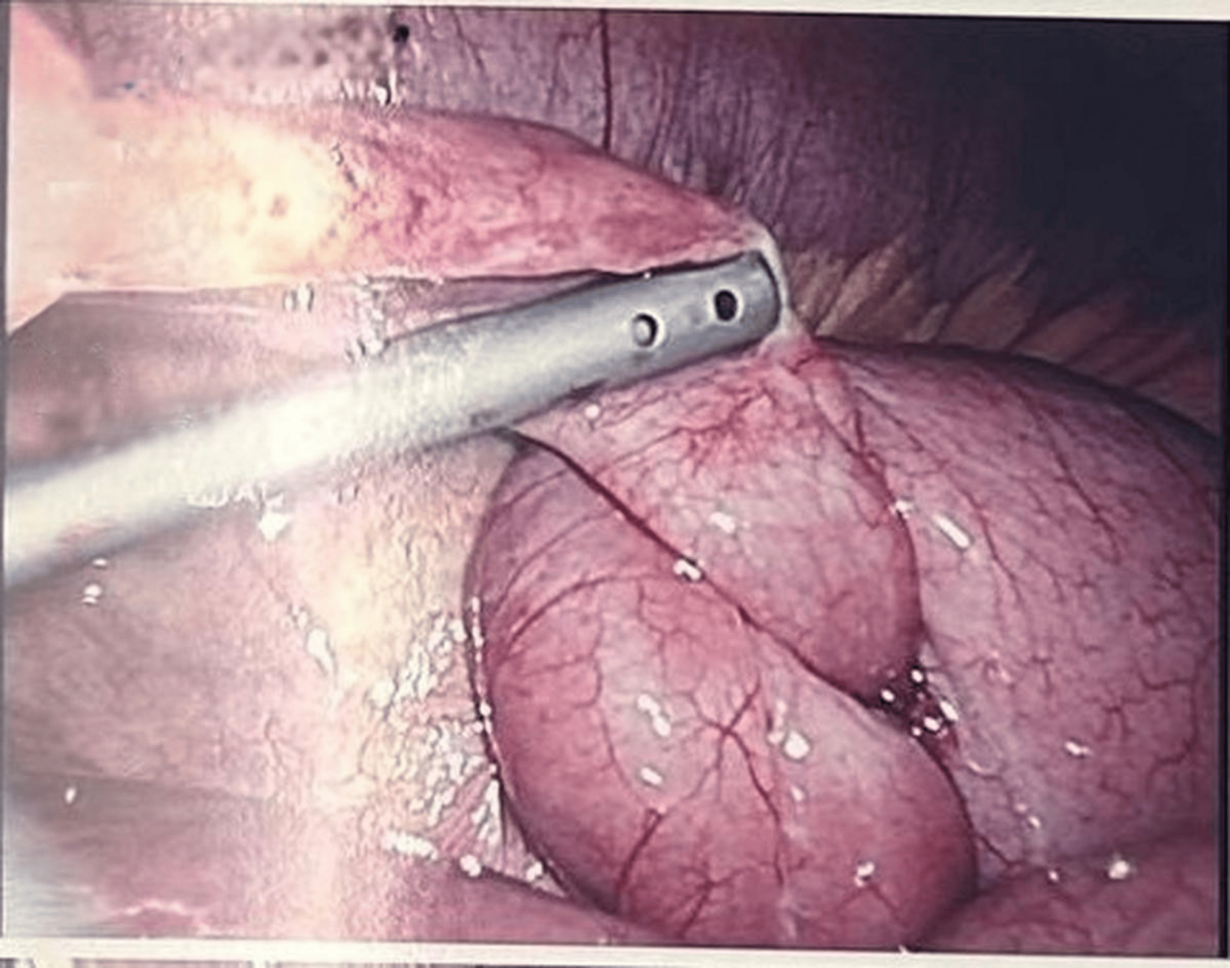
Intraoperative image showing the patent duct attached to the umbilicus and causing small bowel obstruction.

**Figure 2:**
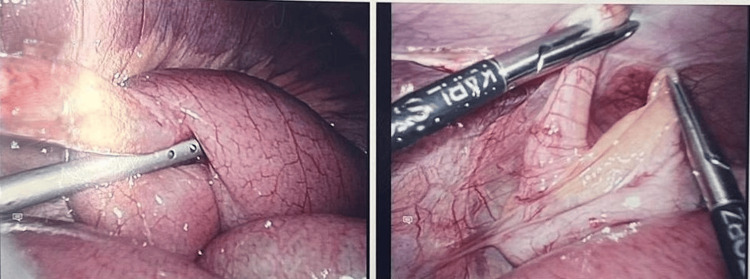
Intraoperative image showing twisted, viable small bowel loop secondary to the omphalomesenteric duct. The appendix appears grossly normal.

Due to ongoing pain and clinical concern for appendicitis despite conservative management, the decision was made to proceed with a diagnostic laparoscopy with the intent to perform an appendectomy. Intraoperatively, a fibrous tubular band consistent with a patent omphalomesenteric duct was identified, extending from the distal ileum to the undersurface of the umbilicus, as shown in Figure 1. A loop of small bowel had become twisted around this cord, creating a closed-loop obstruction; however, the entrapped bowel segment was still viable, and the appendix appeared macroscopically normal (Figure 2).

**Figure 3:**
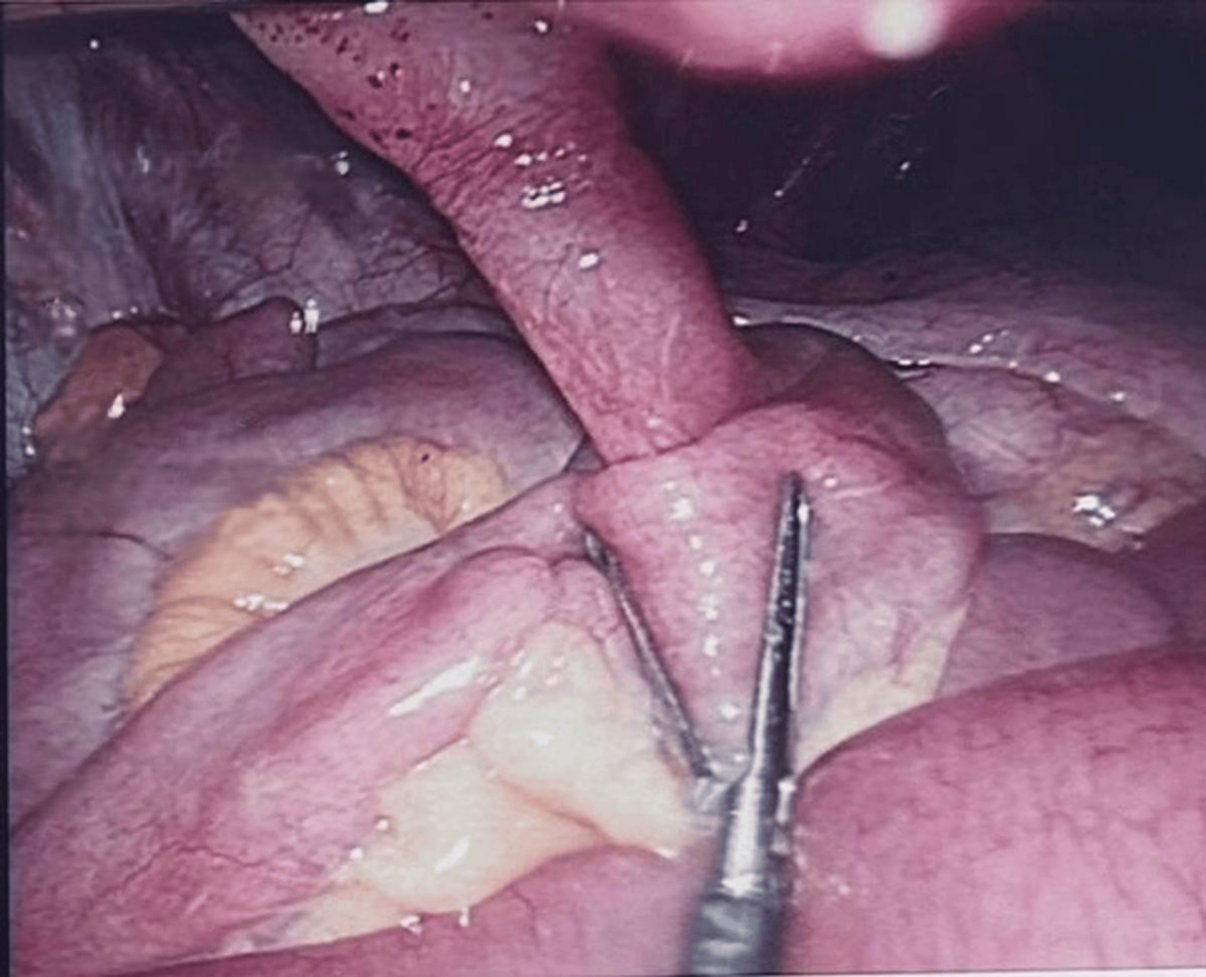
Intraoperative image after untwisting the bowel, showing the umbilicus and viable bowel.

**Figure 4:**
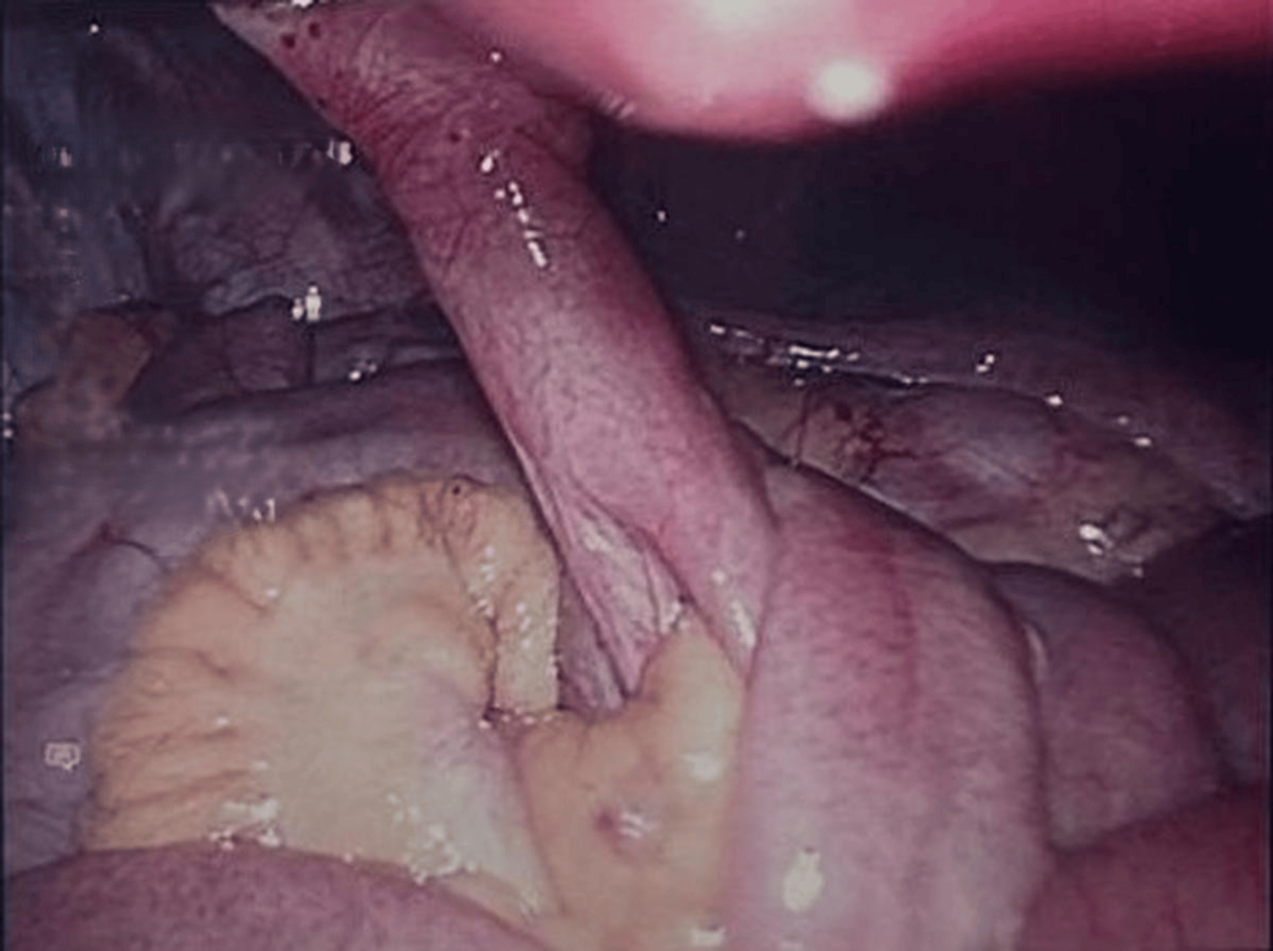
Intraoperative image showing the fibrous band consistent with a patent omphalomesenteric duct, attached to both the small bowel and the umbilicus, following detorsion of the bowel.

The twisted bowel was carefully untangled, as illustrated in Figures 3, 4, and the fibrous duct was divided and resected using a linear stapler. A prophylactic appendectomy was also performed using an endoscopic stapler to avoid future surgical emergencies. Histopathological analysis later confirmed an unremarkable appendix with fibrofatty obliteration, and the resected duct remnant was lined by small intestinal mucosa with focal gastric heterotopia. No dysplasia or malignancy was identified in either specimen.

The patient’s postoperative course was uneventful. Bowel function returned promptly, and he was started on oral intake the next day. He was discharged on the third postoperative day in good condition and remained well at follow-up, with complete resolution of symptoms.

## Discussion

Small bowel obstruction is a common surgical emergency, but an omphalomesenteric duct remnant as the cause is exceptionally rare in adults [4,8]. The omphalomesenteric (vitelline) duct is an embryonic yolk stalk connection to the midgut that normally involutes by the 5th-10th week of gestation [2,4].Failure of complete obliteration can result in a spectrum of anomalies, most commonly Meckel’s diverticulum, and far less commonly a persistent duct or fibrous band [4]. These remnants are present in approximately 2% of people (usually as Meckel’s diverticulum), but they seldom cause symptoms in adulthood [1,9].In fact, vitelline duct anomalies typically manifest in childhood, e.g., intestinal obstruction, gastrointestinal bleeding, and umbilical drainage, and are often asymptomatic in adults [10].

Our patient represents a rare delayed presentation of this congenital anomaly. The absence of prior abdominal surgery is notable, as congenital bands like a persistent omphalomesenteric duct can be considered in patients with a “virgin abdomen” presenting with obstruction, and what makes it challenging is that our patient never had any umbilical problem or any issues with the bowel in the past.

Intraoperatively, the patent duct acted as a fibrous band, creating a closed-loop obstruction by twisting a bowel loop around it. While such remnants can cause obstruction via intussusception, volvulus, or internal herniation, our patient’s bowel remained viable. In contrast, a similar case in the literature resulted in gangrene and required bowel resection, highlighting the potential severity of this condition [7].

Preoperative diagnosis of an omphalomesenteric duct remnant is often challenging due to non-specific imaging findings. Ultrasound was non-diagnostic in our case, and although CT may identify anomalous bands, diagnostic laparoscopy provided both definitive diagnosis and treatment [11].This minimally invasive approach aligns with existing reports supporting laparoscopic management of such anomalies [11].Prophylactic appendectomy was also performed to prevent future diagnostic uncertainty.

Once an omphalomesenteric remnant is identified and causes symptoms, surgical resection is the recommended treatment [11,12].

## Conclusions

This case highlights a rare cause of acute abdomen and small bowel obstruction in an adult: a persistent omphalomesenteric duct, a vestigial remnant of embryologic development. The patient’s presentation closely resembled acute appendicitis; however, diagnostic laparoscopy revealed an uncommon congenital band as the underlying cause. Prompt surgical intervention resulted in symptom resolution and prevented further complications. Although exceedingly rare, a patent omphalomesenteric duct should be considered in the differential diagnosis of small bowel obstruction, particularly in young adults with no prior abdominal surgery. This case also underscores the value of minimally invasive surgery in both diagnosing and treating atypical causes of obstruction. Greater awareness of such anomalies can support early recognition and effective management, ultimately leading to improved patient outcomes.
